# Preclinical evaluation of the PI3K/Akt/mTOR pathway in animal models of multiple sclerosis

**DOI:** 10.18632/oncotarget.23862

**Published:** 2018-01-03

**Authors:** Santa Mammana, Placido Bramanti, Emanuela Mazzon, Eugenio Cavalli, Maria Sofia Basile, Paolo Fagone, Maria Cristina Petralia, James Andrew McCubrey, Ferdinando Nicoletti, Katia Mangano

**Affiliations:** ^1^ Department of Biomedical and Biotechnological Sciences, University of Catania, Catania, Italy; ^2^ IRCCS Centro Neurolesi Bonino-Pulejo, Messina, Italy; ^3^ Department of Educational Sciences, University of Catania, Catania, Italy; ^4^ Department of Microbiology and Immunology, Brody School of Medicine, East Carolina University, Greenville, NC, USA

**Keywords:** multiple sclerosis, mTOR, autoimmunity, bioinformatics

## Abstract

The PI3K/AKT/mTOR pathway is an intracellular signalling pathway that regulates cell activation. proliferation, metabolism and apoptosis. Increasing body of data suggests that alterations in the PI3K/AKT/mTOR pathway may result in an enhanced susceptibility to autoimmunity. Multiple Sclerosis (MS) is one of the most common chronic inflammatory diseases of the central nervous system leading to demyelination and neurodegeneration.

In the current study, we have firstly evaluated in silico the involvement of the mTOR network on the generation and progression of MS and on oligodendrocyte function, making use of currently available whole-genome transcriptomic data. Then, the data generated in silico were subjected to an ex-vivo evaluation. To this aim, the involvement of mTOR was validated on a well-known animal model of MS and *in vitro* on Th17 cells.

Our data indicate that there is a significant involvement of the mTOR network in the etiopathogenesis of MS and that Rapamycin treatment may represent a useful therapeutic approach in this clinical setting. On the other hand, our data showed that a significant involvement of the mTOR network could be observed only in the early phases of oligodendrocyte maturation, but not in the maturation process of adult oligodendrocytes and in the process of remyelination following demyelinating injury.

Overall, our study suggests that targeting the PI3K/mTOR pathway, although it may not be a useful therapeutic approach to promote remyelination in MS patients, it can be exploited to exert immunomodulation, preventing/delaying relapses, and to treat MS patients in order to slow down the progression of disability.

## INTRODUCTION

Multiple Sclerosis (MS) is one of the most common chronic inflammatory diseases of the central nervous system leading to demyelination and neurodegeneration.

The PI3K/Akt/mTOR signalling pathway is strictly involved in T cell responses. Both mTORC1 and mTORC2 are activated within minutes after TCR stimulation and the magnitude of mTOR activation is directly correlated with the duration of interaction between T cells and dendritic cells. Increasing body of data suggests that alterations in the PI3K/Akt/mTOR pathway may result in enhanced susceptibility to autoimmunity [1, 2]. Beside regulating immune responses, the PI3K/Akt/mTOR has also been proven to be involved in several other functions including, chemoresistance, aging and host response to viral infections [3–8].

Therefore, drugs targeting the PI3K/Akt/mTOR pathway are currently under extensive investigation for their possible use in different therapeutic settings, and they have been approved for the treatment of certain forms of cancer, prevention of allograft rejection, for tuberous sclerosis complex-associated renal angiomyolipoma and subependymal giant cell astrocytoma [9–12].

Of note, approval of PI3K/Akt/mTOR inhibitors for immunoinflammatory and autoimmune diseases is so far lacking, although large and independent clinical studies have shown clear-cut efficacy of rapamycin in patients with non-infectious posterior uveitis.

The identification of novel disease indications for approved drugs, a.k.a. drug repositioning, offers several advantages over traditional drug development [13]. Indeed, drug discovery is generally protracted and costly, taking on average approximately 15 years and over $1 billion to develop and bring a novel drug to the market [14]. A large part of drug development costs are employed during early development and toxicity testing, with more than 90% of drugs failing to move beyond these early stages [15]. The repositioning of drugs already approved for human use cuts the costs and risks associated with early stages of drug development, and offers shorter paths to approval for novel therapeutic indications.

Preclinical and clinical studies anticipating a beneficial role for mTOR inhibitors in patients with multiple sclerosis [16] have prompted us to evaluate the potential use of Rapamycin in the context of MS. The study followed a multi-tier approach, based on in silico, *in vitro* and *ex vivo* analysis. The first in silico approach was aimed at evaluating the involvement of the mTOR network on the generation and progression of MS. Then, we evaluated whether genes modulated by Sirolimus were among those observed to be altered in the disease. The hypothesis underlying this approach is that if a drug affects the expression of genes oppositely modulated in a disease, that drug has the potential to be used for the treatment of that disease.

Next, we wanted to evaluated the involvement of the mTOR network on oligondendrocyte function, in order to ascertain whether treatment with drugs targeting the PI3K/Akt/mTOR pathway may be useful to promote the remyelination process, so to reverse disability in MS patients.

Finally, the data generated in silico were subjected to an ex-vivo evaluation. To this aims the involvement of mTOR was validated first on a well-known and established animal model of MS in the mouse, the MOG-induced EAE model. Finally, given the role of Th17 cells in the pathogenesis of EAE/MS, we determined whether mTORC1 inhibition via Rapamycin treatment on CD4 T cells upon Th17 conditions, was able to modulate the expression levels of the genes previously identified in the in silico analysis.

## MATERIALS AND METHODS

### *In silico* analysis

#### Generation of the MS “Disease gene signature” and of Rapamycin “Drug regulated genes”

Expression datasets for disease and drug effects were obtained from the NCBI Gene Expression Omnibus (GEO, http://www.ncbi.nlm.nih.gov/geo/). MESH terms “Multiple Sclerosis” and “Rapamycin” were used to identify potential datasets of interest and GSE29606 and GSE38645 were chosen for the analysis. The GEO2R web application was used to identify differentially expressed genes. In cases where multiple microarray probes mapped to the same NCBI GeneID, we chose the probes which showed the maximum variance. On each data set, we performed moderate t statistics to generate a list of up-regulated and down-regulated genes. We used a threshold of *p* < 0.05 and fold change > 1.5. The list of significantly up and down regulated genes for each comparison were considered for further analysis.

### Generation of the mTOR “Regulatory molecular network”

Highly complex phenotypes arise from a relatively restricted set of gene families connected by a tightly regulated network of interactions. The STRING database (http://string-db.org/) allows to have access to a global view of all the available interaction data by creating large networks, which captures the current knowledge on the functional modularity and interconnectivity of genes in a cell. The majority of associations generated with STRING derives from predictions which are based on analyzing genomic information or from transferring associations/interactions among organisms. All associations are provided with a confidence score that represents a rough estimate of how likely a given association describes a functional linkage between two proteins. For the current study, the search term “MTOR” was used to generate a network, using a confidence score of 0.7 and by including no more than 20 interactors per gene.

### Dataset selection and analysis of oligodendrocyte damage and remyelination processes

Expression datasets were obtained from NCBI Gene Expression Omnibus (GEO, http://www.ncbi.nlm.nih.gov/geo/). GSE32645 and GSE48872 were chosen for the analysis. From GSE32645, we selected the three active MS lesions samples (one fulminant active lesion and two chronic active lesions), and the three cortex samples from controls without brain pathology. Complete demographic data of patients and controls can be obtained from the relative publication [17]. GSE48872 included gene expression profiles from neonatal oligodendrocyte precursors (nOPCs) and adult OPCs isolated from the brain of postnatal (day 1 to day 5) and 2-month-old mice, while adult OPCs in demyelinating conditions (activated aOPCs) were isolated from the brain of mice previously treated for 5 weeks with cuprizone (0.2%). Adult oligodendrocytes (OLs) were obtained from brains of 2-month-old mice [18]. The GEO2R web application was used to identify differentially expressed genes. In cases where multiple microarray probes mapped to the same NCBI GeneID, we chose the probes which showed the lowest *p* value. On each data set, we performed a Student’s *T* test to generate a list of up-regulated and down-regulated genes. We used a threshold of *p* < 0.05 and fold change > 2. The list of significantly up and down regulated genes for each comparison were considered for the gene enrichment analysis using the Chi-square test with Yates’ correction using the mTOR pathway as background. A *p* value < 0.05 was considered to be statistical significant.

### Gene enrichment analysis

In order to evaluate the significance of gene-term enrichment using the mTOR “Regulatory Molecular Network” or the Rapamycin “Drug regulated genes” as background, a Chi-square test with Yates’ correction was performed for the selected genes. A *p* value < 0.05 was considered to be statistical significant.

### *Ex vivo* and *in vitro* analysis

#### Animals

Eight to 10 weeks old female C57BL⁄6 mice will be purchased from ENVIGO RMS srl (San Pietro al Natisone, Udine, Italy). The animals were kept at the animal facility of the Department of Biomedical and Biotechnological Sciences, Section of General Pathology, Catania, Italy. They were kept under standard laboratory conditions (non-specific pathogen-free) with free access to food (Harlan Global Diet 2018) and water and were allowed to adapt at least one week to their environment before commencing the study. Automatically controlled environmental conditions were set to maintain temperature at 20 – 24°C with a relative humidity (RH) of 30 – 70% ,10-30 air changes /hr and a natural dark:light cycle. Protection of animals used in the experiment is in accordance with Directive 86/609/EEC, enforced by the Italian D. Lgs 26/2014.

### Induction of MOG-induced EAE in C57BL⁄ 6 mice

MOG _35-55_ was synthetized by Genemed synthesis (San Francisco CA). The mice were immunized with 200 ug MOG emulsified in CFA with 1 mg of Mycobacterium tuberculosis H37RA (Difco, Detroit, MI, USA) to make a 1:1 emulsion. Each mouse received subcutaneous injections of 200 ml emulsion divided among two sites draining into the axillary lymphnodes. Pertussis toxin (Calbiochem, Nottingham, UK) was used as a co-adjuvant and was administered i.p. at the dose of 200 ng/mouse on day 0 and 2 post immunization. The mice were observed every day by measuring their body weights and clinical signs of EAE. The clinical grading were carried out by an observer unaware of the treatment: 0 = no sign of disease; 0.5 = partial tail paralysis; 1 = tail paralysis; 1.5 = tail paralysis + partial unilateral hindlimb paralysis; 2 = tail paralysis + hindlimb weakness or partial hindlimb paralysis; 2.5 = tail paralysis + partial hindlimb paralysis (lowered pelvi); 3 = tail paralysis + complete hindlimb paralysis; 3.5 = tail paralysis + complete hindlimb paralysis + incontinence; 4 = tail paralysis + hindlimb paralysis + weakness or partial paralysis of forelimbs; 5 = moribund or dead.

### *Ex vivo* restimulation with MOG _35-55_

At the 14th day after EAE induction, spleens were harvested from C57BL/6 mice. Cell suspensions were prepared by grinding the organs with the plunger of a 5 ml disposable syringe and suspending them in RPMI 1640 medium supplemented with 10% fetal calf serum, 2 mM glutamine, and 50 mg/ml of penicillin/streptomycin (complete medium). Splenocytes were treated with an ACK Lysis Buffer (Invitrogen, Monza, Italy) to remove red blood cells. Cells were pelleted and washed twice with PBS. Cells were resuspended at 2 × 10^6^ cells/mL in complete medium and re-restimulated with 40 µg/mL of MOG or Concanavalin A (ConA; 10 μg/ml, Sigma Aldrich St. Louis MO, USA), and treated with increasing concentrations of BEZ-235, PX-866 and Rapamycin. Stock solutions were prepared in 100% dimethyl sulfoxide (DMSO) (Sigma-Aldrich). Subsequent dilutions and controls were prepared to account for the inclusion of DMSO in the stock solution. The effect of test compounds on MOF-induced cell proliferation was measured using a BrdU (bromodeoxyuridine) assay kit (Calbiochem). The assay was performed as per the manufacturer›s protocol and proliferation was detected using a spectrophotometric measurement of absorbance at 450 nm.

### Supernatant TNF-alpha levels

For cytokine assays, harvested cells were plated in triplicate in 24-well microtiter plates at a concentration of 2 × 10^6^ cells/well, in the same medium, stimulated with MOG _35-55_ (40 μg/ml) and incubated for 48h. At the end of the incubation period, supernatants were also collected for subsequent determination of TNF-alpha by ELISA (R&D systems, Minneapolis, MN, USA).

### *In vitro* effects of Rapamycin

Murine CD4+ CD25- T cells were magnetically isolated from wild-type mice. Cells were activated by adding 0.3 mg/mL hamster-anti-mouse CD3 and 0.5 mg/mL hamster-CD28 antibodies to cells in wells pre-coated with 0.3 mg/mL goat-anti-hamster IgG. Th17-polarization was induced using 3 ng/mL recombinant human TGF-β1 and 30 ng/mL recombinant mouse IL-6. 10 nM rapamycin, or vehicle control (DMSO) were added to cultures at the time of activation. Cells were harvested at 18 and 72 hours post-activation and RNA isolated from each sample for subsequent qRT-PCR analysis.

Total RNA was extracted using Trizol reagent (Invitrogen, Monza, Italy) according to the manufacturer’s protocol. Reverse transcription reactions were performed using retro-transcription reagents from Roche. Real-time PCR analyses were carried out using primers in house designed or downloaded from PrimerBank (https://pga.mgh.harvard.edu/primerbank/).

### Statistical analysis

Data are shown as Mean±S.D. of at least three biological replicates, each one being performed as technical triplicate. Statistical analysis was performed using either the Student’s *T* test or Mann-Whitney *U* test based on the results obtained from the Shapiro-Wilk and Kolmogorov-Smirnov Normality Test. The GraphPad Prism was used for the statistical analysis and the generation of the graphs. Fisher’s Inverse χ2 test was used as integrative analysis for rapamycin effects on Th17 cells. It computes a combined statistic from the *P*-values obtained from the individual datasets,

$$\text{s}=-\log\sum_{i=0}^{n}\left(\mathrm{pi}\right)$$

which follows a χ2 distribution with 2/degrees of freedom under the null hypothesis.

## RESULTS

### Generation of the mTOR network

**Figure 1:**
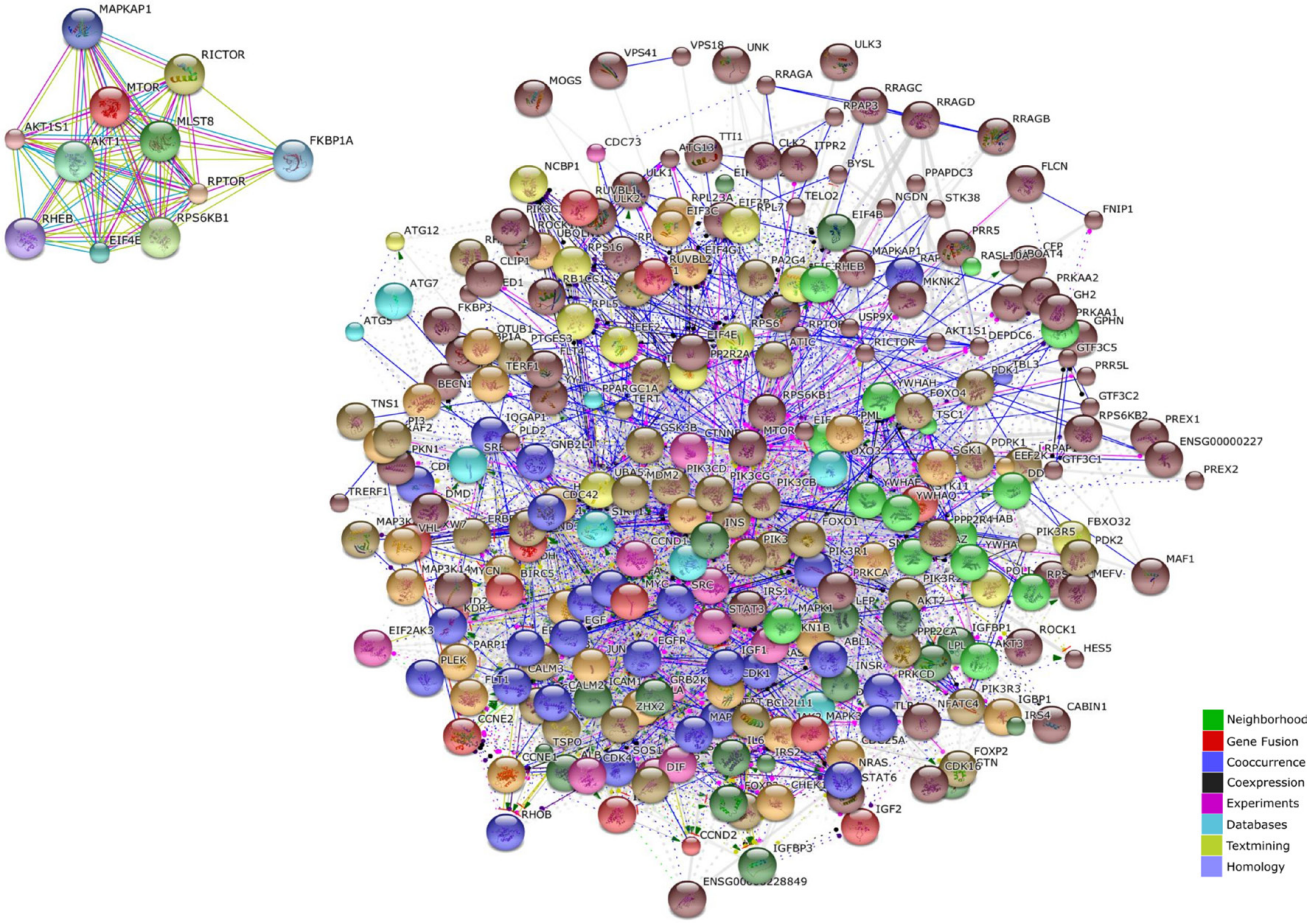
mTOR regulatory molecular network (obtained in STRING DB)

**Figure 2:**
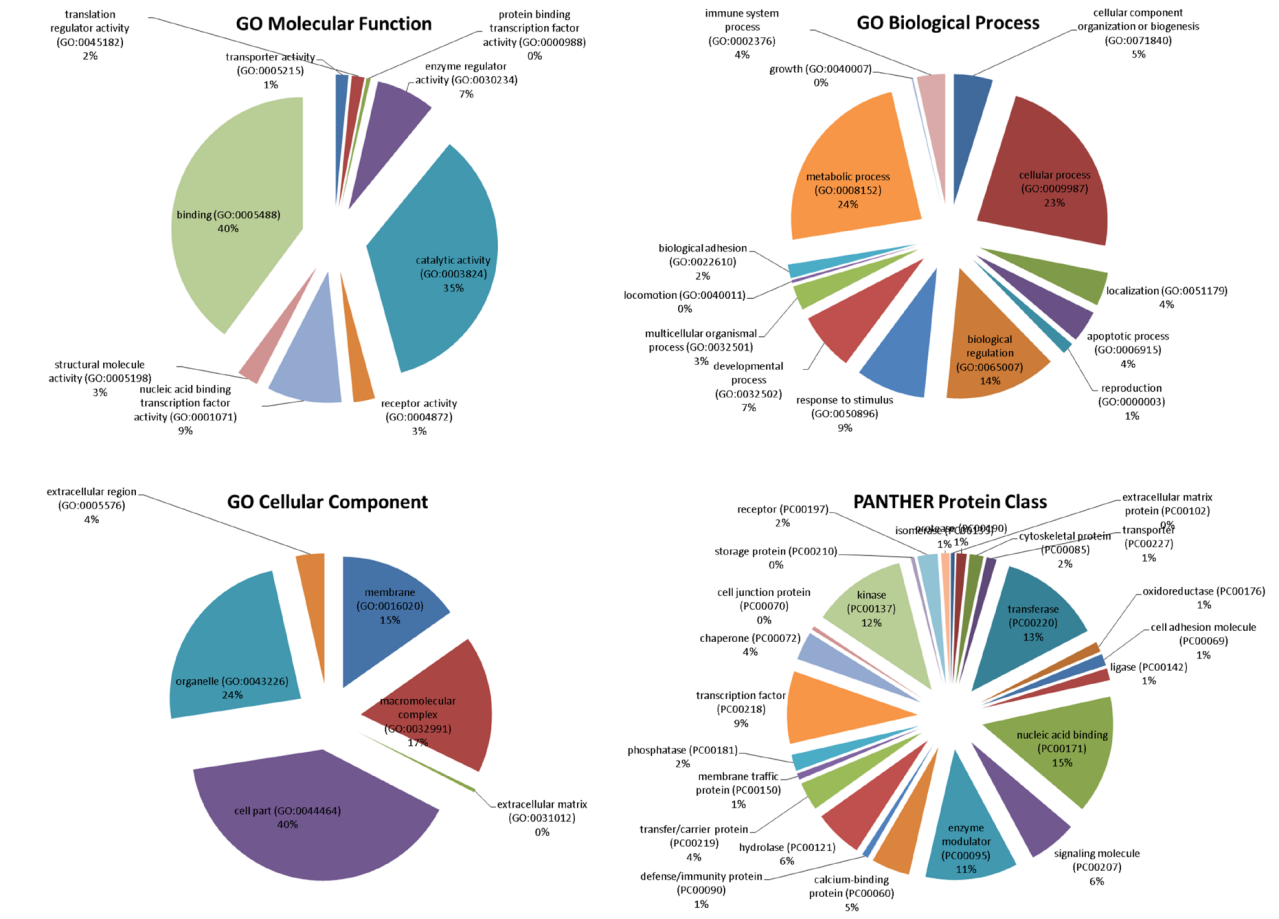
mTOR regulatory molecular network ontology (performed with PANTHER software)

The mTOR network generated by STRING included 318 unique genes (Figure 1). Gene Ontology (GO) analysis identified as the top 3 Molecular Functions: binding activity (GO:0005488); catalytic activity (GO:0003824); and nucleic acid binding transcription factor activity (GO:0001071). Binding activity (GO:0005488) included: protein binding (GO:0005515)(51.10% of the genes); nucleic acid binding (GO:0003676) (34.50% of the genes); calcium ion binding (GO:0005509)(6.90% of the genes); calcium-dependent phospholipid binding (GO:0005544) (5.70% of the genes); and chromatin binding (GO:0003682) (1.70% of the genes) (Figure 2).

Catalytic activity (GO:0003824) included: transferase activity (GO:0016740) (39.00% of the genes); hydrolase activity (GO:0016787) (29.90% of the genes); enzyme regulator activity (GO:0030234) (17.10% of the genes); ligase activity (GO:0016874) (5.50% of the genes); oxidoreductase activity (GO:0016491) (3.70% of the genes); isomerase activity (GO:0016853) (3.70% of the genes); and lyase activity (GO:0016829) (1.20% of the genes (Figure 2)).

Among the top enriched Biological Processes, we found: cellular process (GO:0009987); metabolic process (GO:0008152); and biological regulation (GO:0065007), followed by response to stimulus (GO:0050896); developmental process (GO:0032502); cellular component organization or biogenesis (GO:0071840); immune system process (GO:0002376); multicellular organismal process (GO:0032501); apoptotic process (GO:0006915); localization (GO:0051179) and biological adhesion (GO:0022610) (Figure 2).

The most represented Protein Classes in the mTOR network were: nucleic acid binding (PC00171) proteins, transferases (PC00220), kinases (PC00137), enzyme modulators (PC00095) and transcription factors (PC00218). Less represented protein classes were: signaling molecules (PC00207), calcium-binding proteins (PC00060), hydrolases (PC00121), chaperones (PC00072), transfer/carrier proteins (PC00219), cytoskeletal proteins (PC000085) and phosphatases (PC00181) (Figure 2).

### Involvement of the mTOR network in MS

**Figure 3:**
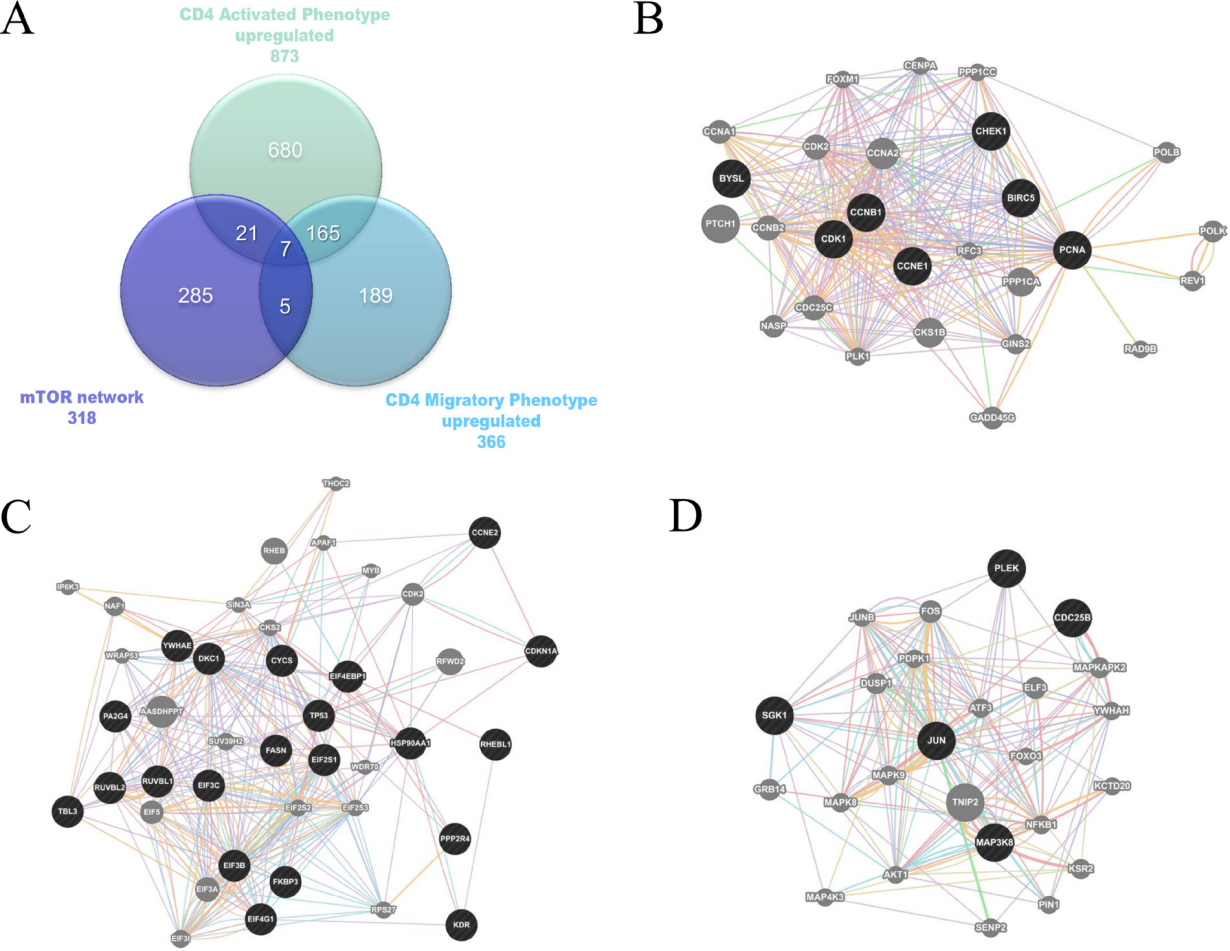
(**A**) Venn Diagram showing shared genes between the mTOR network and significantly upregulated genes in the GSE38645 dataset; (**B**) Network constructed on the 7 genes shared among the mTOR network, the CD4 Activated and the CD4 Migratory Phenotypes; (**C**) Network constructed on the 21 genes shared among the mTOR network and the CD4 Activated Phenotype; (**D**) Network constructed on the 5 genes shared among the mTOR network and the CD4 Migratory Phenotype.

The mTOR network was used for comparison with the upregulated genes involved in MS development as obtained from GSE38645. For the latter, total RNA was extracted from sorted *ex vitro* myelin specific CD4+T cells (*activated phenotype*) or isolated from the spleen (*migratory phenotype*). Myelin specific activated and migratory CD4+ T cells were defined by 873 and 366 up-regulated genes, respectively, with 172 genes being shared between the two groups (Figure 7; Table 1). When comparing these genes with those belonging to the mTOR pathway, we found that 28 were in common with the activated phenotype and 12 with the migratory phenotype. Chi square test revealed a strong significant enrichment (*p* < 0.0001) in both cases. 7 genes shared among the mTOR network, the CD4 Activated and the CD4 Migratory Phenotypes (Figure 3).

**Table 1:** Involvement of rapamycin in multiple sclerosis

Rapamycin Downregulated genes (*n =* 217) vs.		Genes in common	*P* Value
	CD4 Activated Phenotype Upregulated genes (*n =* 873)	AIMP1, CKDK, CENPM, KIF20A, LMNB1, MCM4, ME2, MKI67,PSMD7, TFRC, TUBA4A, UHRF1	***P = 0.0418***
	CD4 Migratory Phenotype upregulated genes (*n* ***=*** 366)	KIF20A, LMNB1, MCM4, MKI67, S100A11, UHRF1	*P* = 0.0801
	Common genes between the CD4 Activated and Migratory Phenotype (*n* ***=*** 172)	KIF20A, LMNB1, MCM4, MKI67,UHRF1	***P = 0.0036***
Rapamycin Upregulated genes (*n* ***=*** 297) vs.		Genes in common	*P* Value
	CD4 Activated Phenotype Downregulated genes (*n* ***=*** 546)	CD2, DBP, DDX6, GIT2, IGFLR1,PTPRC,VMP1	*P* = 0.4946
	CD4 Migratory Phenotype Downregulated genes (*n* ***=*** 245)	DBP, IGFLR1,VMP1	*P* = 0.9618
	Common genes between the CD4 Activated and Migratory Phenotype (*n* ***=*** 101)	DBP, IGFLR1, VMP1	*P* = 0.1296

### Rapamycin effects in MS

Differential expressed genes in CD4+ T cells upon Rapamycin treatment were derived from the GSE29606 dataset. 217 and 297 genes were found respectively downregulated and up-regulated by Rapamycin in anti-CD3/CD28 stimulated CD4+CD25- T cells (Table 1).

Rapamycin was associated to the downregulation of 12 (*p* = 0.029) and 6 (*p* = 0.075) genes implicated in the activated and migratory phenotype, respectively (Table 1). No significant enrichment was observed for the genes up-regulated by Rapamycin treatment (Table 1).

### Involvement of the mTOR pathway in MS cortical lesions

Gene expression analysis of cortical MS lesion, revealed that 126 and 401 genes are significantly up- and down-regulated, respectively, as compared to control brains. Among these genes, 3 upregulated (*p* = 0.3334) and 9 downregulated (*p* = 0.0486) genes resulted in common to the mTOR network (Table 2). The 3 upregulated genes included BAX, TERT and ERBB2. The 9 downregulated genes included JUN, ATF4, CASP3, CDKN1A, EIF4G1, PIK3R3, PML, STAT3, and YWHAZ. Gene Ontology analysis for the category “Biological Process” of the 9 downregulated genes revealed that the top 3 enriched functions were Metabolic Process (GO:0008152) (24%), Biological Regulation (GO:0065007) (23%), Cellular Process (GO:0009987) (20%) (Table 2).

**Table 2:** Involvement of the mTOR network in oligodendrocyte function

mTOR network (*n =* 318) vs.		Genes in common	*P* Value
	Cortical lesions upregulated genes (*n* ***=*** 126)	*BAX, ERBB2, TERT*	*P* = 0.3334
	Cortical lesions Downregulated genes (*n* ***=*** 400)	*ATF4, CASP3, CDKN1A, EIF4G1, JUN, PIK3R3, PML, STAT3* *YWHAZ*	***P = 0.0486***
	Neonatal OPCs upregulated (*n* ***=*** 2592)	*BCL2, BIRC5, CASP3, CCNB1, CCND1, CCND2, CCND3,* *CCNE1, CDC25B, CDH1, CDH5, CDK1, CDK4, CDKN1A,* *DMD, EGFR, EIF4EBP1, ERBB2, ESR1, ETS1, FLT1, FLT4,* *FOS, FOXP2, GNB2L1, HES5, ID2, IGF1, IGF2, IGFBP1,* *IGFBP3, IRS1, ITPR2, LPL, MAGED1, MAP3K8, MKNK2,* *MYC, MYCN, NFATC4, PAX6, PDK1, PIK3R3, PPARGC1A,* *PRKCD, RAC2, RPS16, RRAGD, SMAD3, TNS1, TRERF1,* *ULK2*	***P < 0.0001***
	Neonatal OPCs downregulated (*n* ***=*** 1714)	*BCL2L1, CALM1, CRTC2, DIS3L2, ERBB3, FBXO32, FLT3, HDAC9, JAK3, KDR, MAPK14, MAPK3, NFKB1, PDK2,* *PIK3C3, PPARG, PRKCA, PRR5L, RAP1A, RHOB, RPS6KA1,* *RPS6KB2, SIRT7, SREBF1, STK38, TSC2, YWHAQ*	*P* = 0.0716
	Adult OLs upregulated (*n* ***=*** 251)	*CDKN1A, STK38, TNS1*	*P* = 0.8512
	Adult OLs downregulated (*n* ***=*** 432)	*CDC25B, CDH1, EGFR, ID2, IGF2, MYOD1*	*P* = 0.6944
	Activated aOPCs upregulated 1167	*ATF4, BCL2, BDNF, CCND1, CDKN1A, EIF4EBP1, FOS,* *IGFBP3, ITPR2,JUN, LPL, MYC, PRKCD, TNF, TSPO*	*P* = 0.5881
	Activated aOPCs downregulated 989	*BCL2L11, IL2, KDR, KIT, KRAS, MYCN, PIK3C3, PIK3CD,* *RAP1A, RPS6KB1, RPS6KB2, RRAGB*	*P* = 0.7915

### Involvement of the mTOR pathway in oligodendrocyte function

First, we wanted to evaluate the involvement of the mTOR network in the developmental process of oligodendrocytes. To this aim, we analyzed the differential transcriptomic features of neonatal oligodendrocyte precursors (nOPCs) as compared to adult oligodendrocyte precursors (aOPCs). We found that nOPCs are characterized by 2592 upregulated genes and 1714 downregulated genes as compared to aOPCs. Among the hyperexpressed genes, 52 belonged to the mTOR network (*p* < 0.0001) while, among the downregulated genes, 27 were in common to the mTOR network (*p* = 0.0716) (Table 2). Gene term enrichment analysis revealed that the 52 genes mainly belonged to the Cellular Process (GO:0009987) (24%), Metabolic Process (GO:0008152) (21%) and Biological Regulation (GO:0065007) (17%) categories (Table 2).

When analyzing the involvement of the mTOR network in adult oligodendrocytes (OLs) development from aOPCs, we found that only 3 and 6 among the upregulated and downregulated genes, belonged to the mTOR network (*p* = 0.8512 and *p* = 0.6944, respectively) (Table 2).

Similarly, the activated aOPCs from cuprizone treated animals showed that among the upregulated and downregulated genes, only 15 and 12 genes belonged to the mTOR network (*p* = 0.5881 and *p* = 0.7915, respectively) (Table 2).

### Effects of PI3K/mTOR inhibitors in MS

In order to validate the data previously obtained in the *in silico* analysis, we immunized C57Bl/6 mice with MOG_35-55_ and at overt disease, splenocytes were collected and re-stimulated with antigenic peptide in the presence of scalar concentrations of the mTORC1 inhibitor, Rapamycin, the PI3K inhibitor, PX-866, and the dual mTOR/PI3K inhibitor, BEZ-235.

The concentrations tested were not toxic to cells, as evaluated upon 72 hours of exposure in healthy mice splenocytes (data not shown).

**Figure 4:**
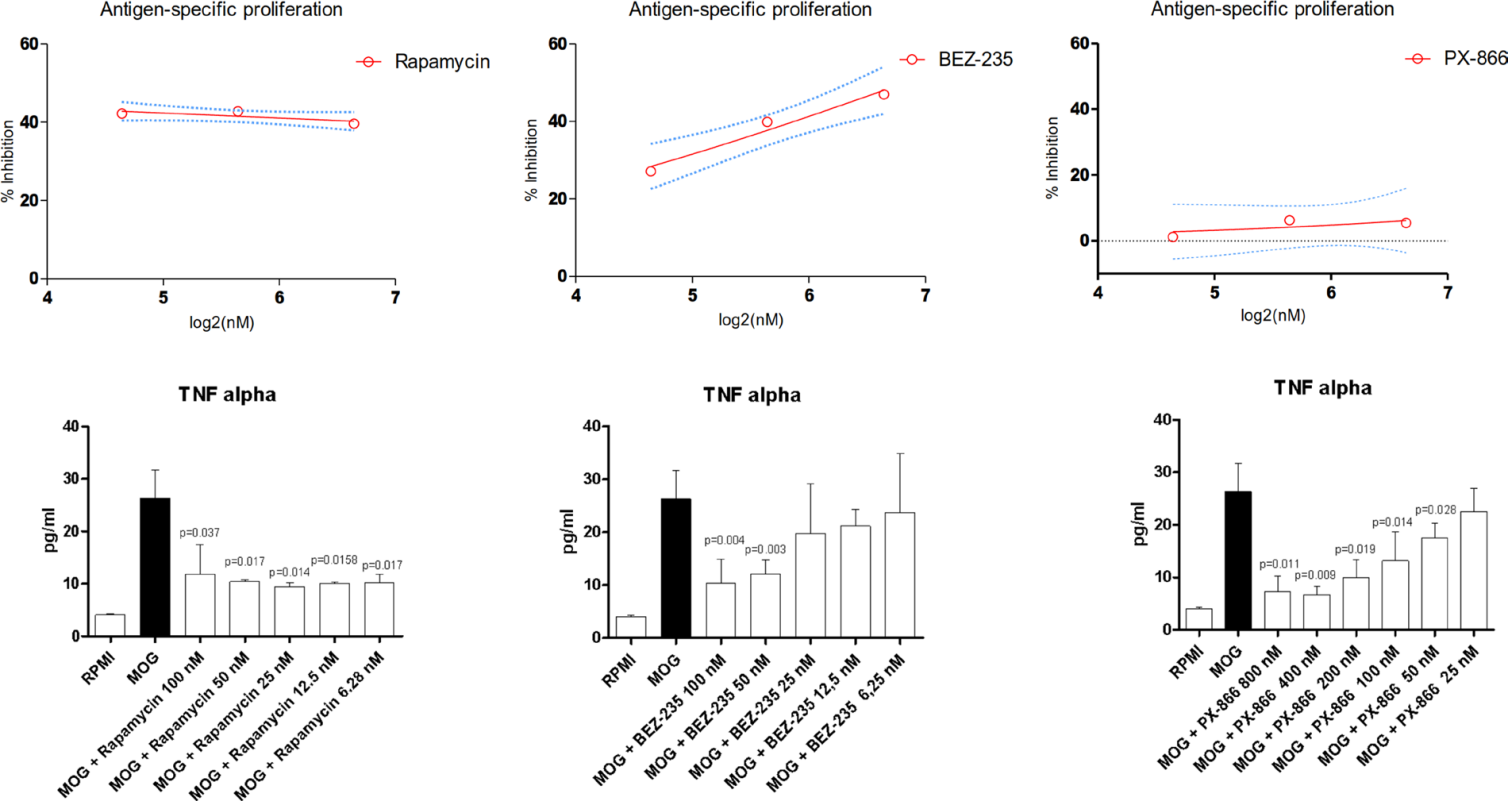
Effects of Rapamycin, BEZ-235 and PX-866 on MOG-specific proliferation and TNF-alpha secretion

Rapamycin was associated to a ∼40% reduction in MOG-specific proliferation in the dose range of 25-100 nM (Figure 4). PX-866 was not effective in reducing the antigen-induced proliferation at concentration up to 100 nM. A modest reduction of ∼20% was observed at the 800 nM concentration.

BEZ-235 exerted a dose-dependent reduction in MOG-specific proliferation, reaching a ∼40% reduction at 100 nM.

We also evaluated the ability of the test compounds to inhibit TNF-alpha production (Figure 4). Rapamycin significantly reduced the levels of TNF-alpha at all the concentrations in the range of 6.25-100 nM, as compared to control treated MOG-stimulated cells (Figure 4). A dose-dependent reduction in cytokine secretion was observed for PX-866 in the dose range of 25-800 nM. No significant reduction was detected for PX-866 at 25 nM concentration (Figure 4). BEZ-235 significantly reduced TNF-alpha levels at 50 and 100 nM concentrations. A reduction, although not significant was however observed also at 6.25–12.5-50 nM (Figure 4).

### Effects of mTORC1 inhibition on T cells under a Th17 polarizing condition

We wanted to evaluate whether mTORC1 inhibition was able to modulate the expression levels of the genes previously identified in the *in silico* analysis (Figure 7B–7D). Given the role of Th17 cells in the pathogenesis of EAE/MS [19], we stimulated murine T cells with anti-CD3/ anti-CD28 antibodies, in the presence of TGF-beta and IL-6, and 10 nM Rapamycin were added to cultures. Gene expression analysis was performed via qRT-PCR at 18 and 72 hours.

**Figure 5:**
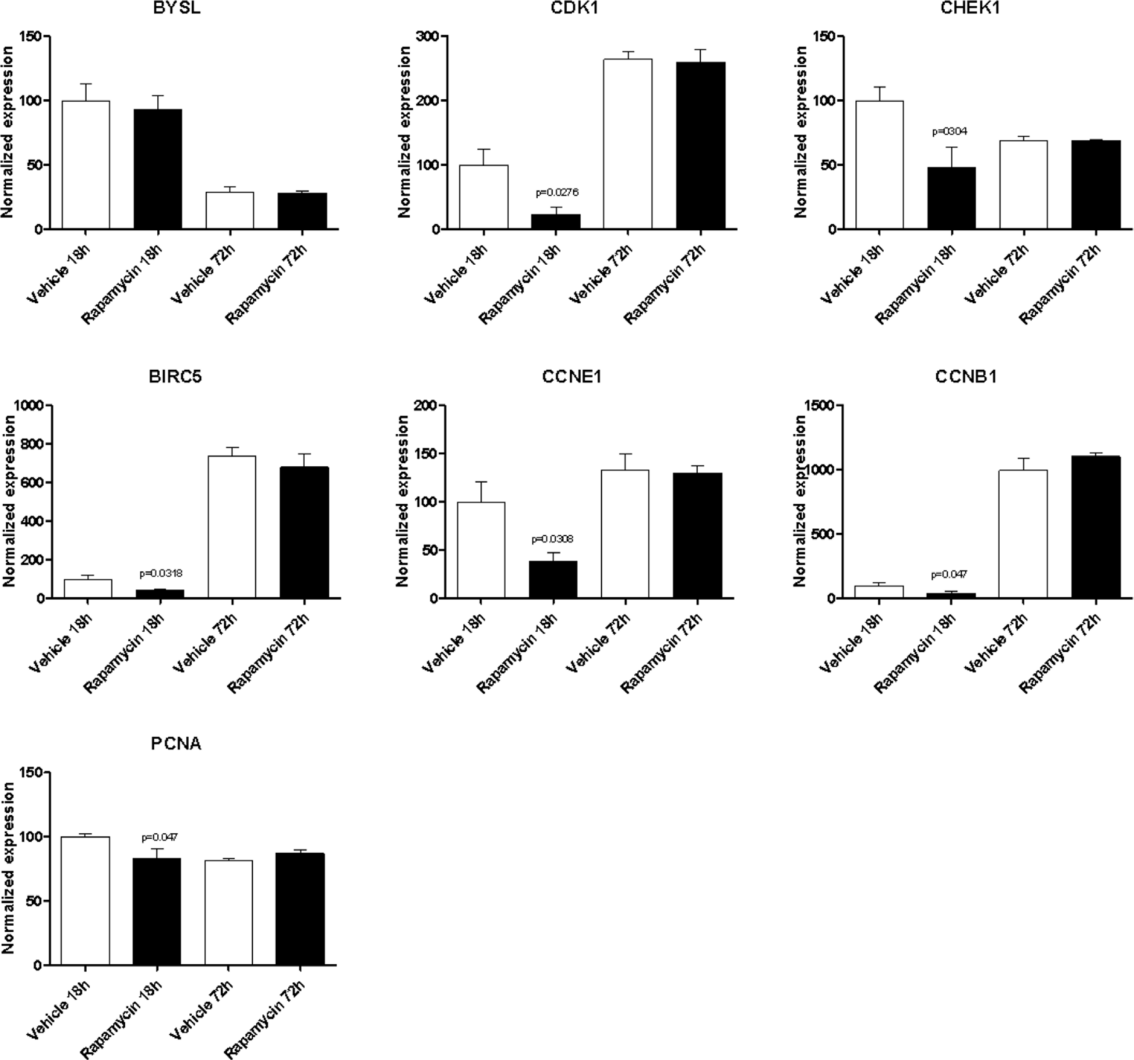
Effect of Rapamycin on expression levels of the genes shared among the mTOR network, the CD4 activated and the CD4 migratory phenotypes in CD4 T cells, as measured by qRT-PCR in Th17 cells

**Figure 6:**
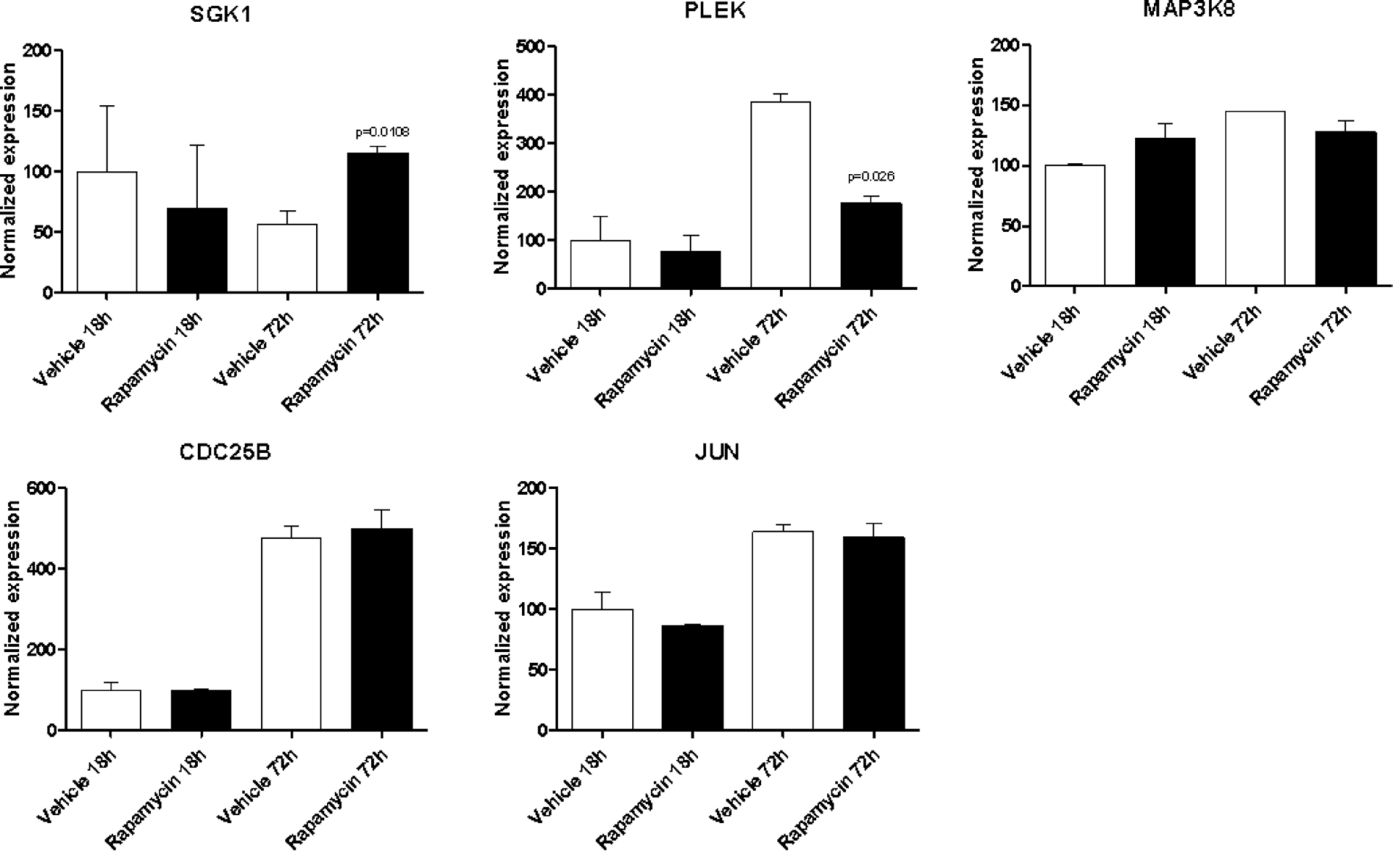
Effect of Rapamycin on the expression levels of the genes shared among the mTOR network and the CD4 migratory phenotype in CD4 T cells, as measured by qRT-PCR in Th17 cells

**Figure 7:**
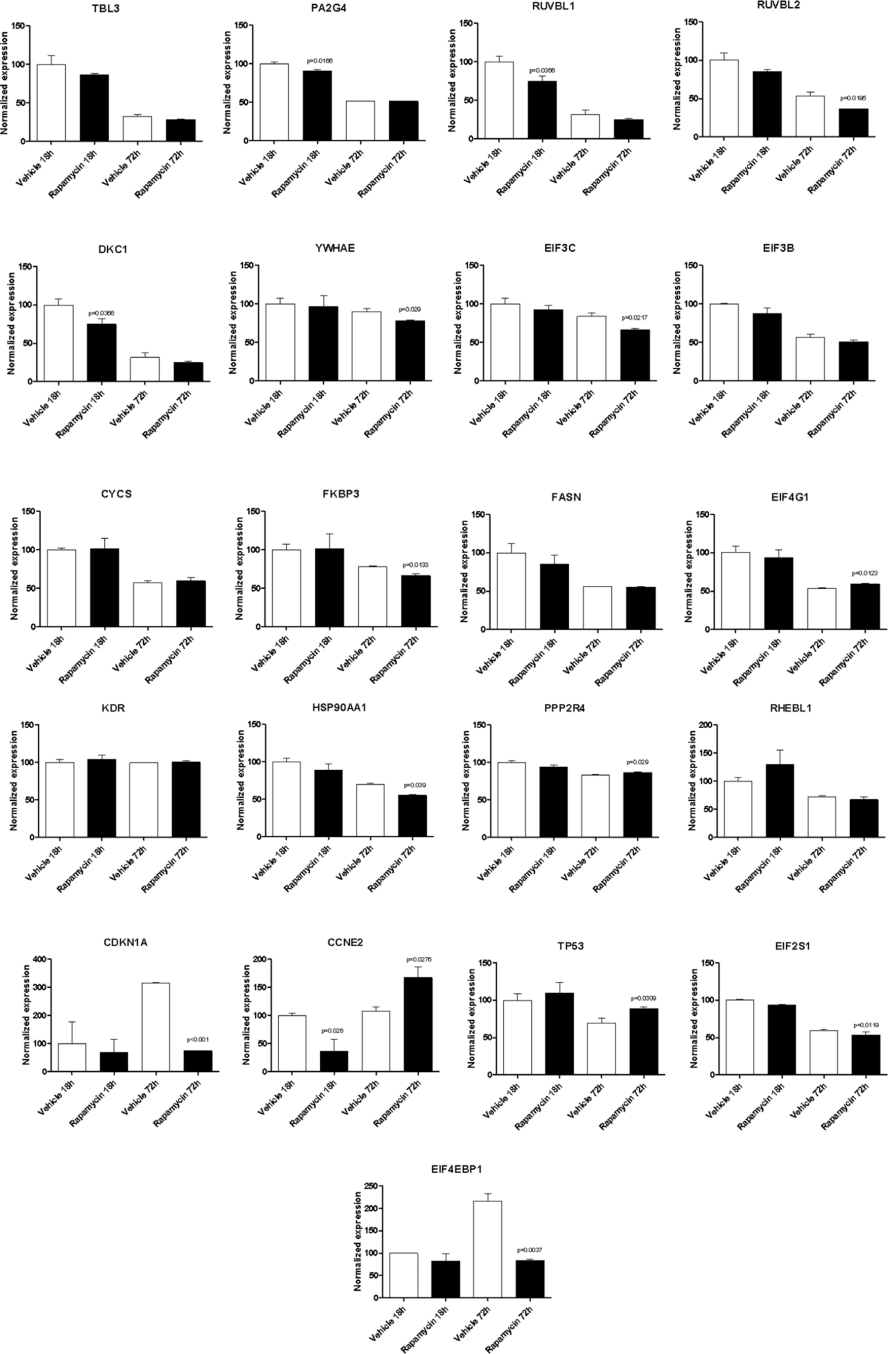
Effect of Rapamycin on the expression levels of the genes shared among the mTOR network and the CD4 activated phenotype in CD4 T cells, as measured by qRT-PCR in Th17 cells

With the exception of BYSL, Rapamycin was able to significantly downregulate the expression of all the genes shared among the mTOR network, the CD4 Activated and the CD4 Migratory Phenotypes in CD4 T cells (a network showing the relationship among these genes is presented in Figure 7B) at 18h of incubation, entailing an overall strong statistical significance (*p* < 0.001 by Fisher Inverse Chi Square) (Figure 5). This effect was not observed after 72h of culture (Figure 5). As regards the effect of Rapamycin on the expression levels of the 5 genes shared among the mTOR network and the CD4 Migratory Phenotype in CD4 T cells (a network showing the relationship among these genes is presented in Figure 6), a significant reduction was observed only for PLEK at 72h (Figure 6). A trend of reduction was observed at 18h for SGK1, although a significant upregulation was detected after 72h. As regards, the 21 genes shared among the mTOR network and the CD4 Activated Phenotype in CD4 T cells (a network showing the relationship among these genes is presented in Figure 7), Rapamycin was able to significantly downregulate PA2G4, RUVBL1, CCNE2 and DKC1 at 18h and RUVBL2, YWHAE, EIF3C, FKBP3, EIF4G1, HSP90AA1, PPP2R4, CDKN1A, EIF2S1 and EIF4EBP1 (Figure 7).

## DISCUSSION AND CONCLUSIONS

MS is one of the most common chronic inflammatory diseases of the central nervous system leading to demyelination and neurodegeneration, with more than 23 million affected peoples. Despite the efforts to understand its etiopathogenesis and to identify effective and tolerable treatment strategies, the majority of patients develop, over the years, important disabilities.

mTOR is a kinase that regulates translation in response to nutrients and growth factors by phosphorylating components of the protein synthesis machinery, including p70 S6K and eukaryotic initiation factor (eIF)-4E binding protein-1 (4EBP-1), allowing eIF-4E to be involved in the assembly of a translational initiation complex [3, 20–25]. mTOR is a key component of the two complexes mTORC1 and mTORC2 and it is at the cross-road of the PI3K/Akt pathway. Indeed, activation of PI3K activates Akt, which localizes in the plasma membrane and exerts a number of downstream effects, including activation of CREB, inhibition p27, localization of FOXO in the cytoplasm, activation PtdIns-3ps, and activation of mTOR. On the other hand, mTORC2 phosphorylates the serine/threonine protein kinase Akt/PKB at a serine residue S473 and serine residue S450. Phosphorylation of the serine promotes Akt phosphorylation at threonine T308 residue by PDK1 and leads to full Akt activation.

Drugs targeting the PI3K/Akt/mTOR pathway are currently under extensive investigation for their possible use as cancer chemotherapeutics and as immunosuppressive agents, but only a limited number of clinical trials is ongoing for the evaluation of their efficacy in the context of immunological disorders. A few independent reports have already shown that the administration of the mTORC1 inhibitor, Rapamycin, is able to modulate the course of the disease in preclinical murine models of MS [26–28]. However the exact role of the mTOR pathway in the development and progression of MS has not yet deeply investigated.

In the current work, we have first undertaken an *in silico* study to evaluate the involvement of the mTOR network on the generation and progression of MS and on oligondendrocyte function, in order to ascertain whether treatment with drugs targeting the PI3K/Akt/mTOR pathway may be useful to affect disease course and to promote the remyelination process, so to reverse disability in MS patients. Next, we wanted to validate the obtained results, *in vitro* and *ex vivo*, using the MOG-induced EAE preclinical model of MS.

Overall, our data indicate that there is a significant involvement of the mTOR network in the ethiopathogenesis of MS and that Rapamycin treatment may represent a useful therapeutic approach in the clinical setting. In particular, Rapamycin appears to be particular effective on activated encephalitogenic T cells rather than on migratory T cells, a data which suggests Rapamycin as a drug useful to treat relapses rather than to prevent them.

Despite the usefulness of this *in silico* approach, there are several drawbacks to bear in mind. First, the therapeutic efficacy of a drug is far more complex than a simple match of expression profiles. Drugs have to reach the appropriate tissue at the appropriate concentration in order to exert an effect, and the route and timing of administration should be careful chosen and sometimes, personalized to the patients, in order to limit undesired side effects. Moreover, compounds may exert different effects on different tissues and organs. On the other hand however, with this approach we could analyze both direct and indirect effects of a drug. Finally, we cannot make informed evaluation whether the drug may find application in all types of a complex disease, such as MS.

*Ex vivo* analysis showed that treatment of MOG-specific T cells from EAE affected mice with the mTOR inhibitor, Rapamycin, and the dual PI3K/mTOR inhibitor, BEZ-235, was able to significantly reduce antigen-specific proliferation, upon MOG restimulation. A much lower effect was on the contrary, observed for the PI3K inhibitor, PX-866, suggesting a secondary role of the PI3K pathway in the regulation of T cell proliferation in response to antigens. Unexpectedly however, all of the three compounds were able to strongly and significantly inhibit the production of the pro-inflammatory cytokine, TNF-alpha, by MOG-restimulated splenocytes from EAE affected mice.

It should be pointed out, that several independent reports have shown that the inhibition of mTOR is associated to a negative feedback loop to Akt. In particular, Wan and collaborators [29] have shown in Rh30 and RD cells that rapamycin-induced increase of Akt phosphorylation was dependent on PI3K. In fact, pretreatment of these cells with the PI3K inhibitor, LY294002, resulted in a reduction of rapamycin-induced increase of Akt activation. On the contrary, the MEK inhibitor, U0126, did not affect the rapamycin-mediated phosphorilation of Akt

Although we have not specifically looked at the activation state of the PI3K/Akt axis following rapamycin treatment, we have observed that there is no substantial improvement in the antigen-specific proliferation and TNF alpha secretion when a dual PI3k/mTOR inhibitor was added to the cells as compared to the selective mTOR inhibitor, Rapamycin. We can speculate that the increased rapamycin-dependent Akt activation could promote the phosphorylation of the Forkhead box family of transcription factors, with their consequent retention in the cytoplasmic compartment and the downregulation of target genes, such as Kruppel-like Factor 2 (KLF2). KLF2 has been reported to control CD62L, C–C Chemokine Receptor 7 (CCR7), and Sphingosine-1-Phosphate Receptor (S1P1R) expression, therefore regulating the homing of lymphocytes to secondary lymphoid tissues [30].

In addition, we show that Rapamycin treatment of murine T cell stimulated under Th17 conditions, is able to significantly inhibit the expression of some of the genes previously identified in the *in silico* analysis. Up to now, only 4 of the genes identified *in silico* have been associated to MS and neurodegeneration, namely BIRC5, SGK1, MAP3K8 and PLEK, and with the exception of MAP3K8, all of these resulted to be modulated by Rapamycin treatment. Moreover, several other genes identified to be shared among the mTOR network and the MS CD4 T cell (i.e., PA2G4, RUVBL1, CCNE2, DKC1, RUVBL2, YWHAE, EIF3C, FKBP3, EIF4G1, HSP90AA1, PPP2R4, CDKN1A, EIF2S1 and EIF4EBP1) resulted significantly downregulated by Rapamycin. These genes included translation factors (EIF4EBP1, EIF3C, EIF4G1 and EIF2S1); centromere-DNA binding factor (DKC1); nucleic acid binding factor (PA2G4); chaperones (YWHAE and HSP90AA1) and chaperone isomerase (FKBP3); and enzyme modulators (PPP2R4, CCNE2 and CDKN1A).

It is well established that the inhibition of the mTOR signaling is able to divert T cell polarization toward an immunoregulatory phenotype. Indeed, mTOR inhibition has been reported to induce the upregulation of FoxP3 and to expand the population of nTregs. On the contrary, an increase in Akt phosphorylation, via deletion of PTEN, is associated to impaired Treg cell polarization. Mtor−/− T cells are characterized by normal TCR-induced activation markers, but are not able to differentiate into either Th1, Th2 or TH17 cells. Moreover, they fail to express the lineage-specific transcription factors, T-bet, GATA3, and RORγt. Along the same lines, Mtor−/− T cells upregulate FOXP3 upon TCR stimulation, even in the absence of polarizing cytokines. It is believed that in T cells the mTOR pathway antagonizes the signaling mediated by SMAD and FOXO, that are involved in the induction of FoxP3. Also, upstream receptors, such as PDL1 and S1PR1, by activating mTOR, are able to inhibit the generation of iTregs. It is likely that mTORC1 and mTORC2 exert diverse effects on T cell polarization. RHEB deficiency, that leads to loss of mTORC1, negatively affects Th1/Th17 differentiation. T cells deficient for RHEB show increased activity of SOCS3, and the silencing of the latter is associated to a restoration of Th1 differentiation. On the other hand, mTORC2 loss, via deletion of RICTOR, is associated to a reduced polarization of T cells toward a Th2 phenotype, without affecting the development of Th17 cells. This effects seems to be mediated by both protein kinase C-theta (PKCθ) and SOCS5 signaling.

On the other hand, it has also been shown that blocking mTOR activity by pharmacological intervention induces T cell anergy *in vitro*, likely interfering with the transduction of the costimulatory signaling of the immunological synapsis (reviewed in [31]).

Although in our experiments on Th17 cells *in vitro*, we have specifically focused on the modulation of genes likely involved in MS development and progression, as determined by the in silico analysis, we were not able to detect a fully polarization toward the Treg phenotype. Indeed, we observed only a moderate upregulation of FoxP3, along with a limited downregulation of IL17F, and no modulation of other Treg markers, including CD121a, IL7R and ARG2 (data not shown).

In the present work, we also wanted to investigate the possible involvement of mTOR in the process of remyelination, in order to evaluate whether the use of drugs targeting the PI3K/Akt/mTOR pathway could exert beneficial effects on the CNS lesions of MS patients.

Remyelination is the process by which new myelin sheaths are generated around axons in the adult central nervous system following injury. Remyelination occurs in the MS lesions but becomes progressively inadequate, thus causing permanent disability in patients. Remyelination occurs in two major steps. The first consists in the colonization of lesions by oligodendrocyte progenitor cells (OPCs), the second in the differentiation of OPCs into myelinating oligodendrocytes that contact demyelinated axons to generate functional myelin sheaths [32].

Current available data on the involvement of mTOR in the myelination process are often discordant. Cell-specific loss of Pten, an endogenous inhibitor of mTOR, in oligodendrocytes and Schwann cells, promotes hypermyelination [33]. Moreover, inhibition of mTOR by Rapamycin interferes with the development of galactocerebroside positive immature oligodendrocytes in purified oligodendrocyte precursor cell (OPC) cultures and also reduces the number of myelinating oligodendrocytes when OPCs are cocultured with dorsal root ganglia neurons [34]. A role for mTOR in oligodendrocyte function has been also hypothesized by Flores and collaborators, who generated transgenic mice overexpressing a constitutive active form of Akt in oligodendrocytes and observed an increased amount of myelin produced per oligodendrocyte without modification of the oligodendrocyte cell number [35]. On the other hand, when Rapamycin is injected in an in vivo model of tuberous sclerosis, myelination was enhanced [36]. In a similar fashion, Rapamycin improved myelination in explant cultures from neuropathic mice by activating autophagic mechanisms [37].

Our data show that a significant involvement of the mTOR network can be observed only in the early phases of oligodendrocyte maturation, i.e. in neonatal OPCs. Little or no contribution of mTOR can be observed in the maturation process of adult oligodendrocytes from adult OPCs, and in the process of remyelination following demyelinating injury.

These observation are concordant with the fact that in active cortical MS lesion, a significant enrichment for the mTOR network can be observed only among the downregulated genes.

Overall, our data suggest that targeting the PI3K/mTOR pathway, although it may not be a useful therapeutic approach to promote remyelination in MS patients, however it can be exploited to exert immunomodulation, preventing/delaying relapses, and to treat MS patients in order to slow down the progression of disability.
